# Illustrations of Syphilitic Disease

**Published:** 1853-05

**Authors:** 


Art. XII.—*Illustrations of Syphilitic Disease.* By Philip Ricord, D. M. P., Chirurgien De L'Hôpital Des Vènériens (Hôpital Du Midi), Chevalier de la Légion D'Honneur et de la Couronne de Chêne, Laurëat de L'Institut de France, (Académie des Sciences), Membre de diveres Sociètes Savantes, Nationales et Etrangres. Translated from the French by Thomas F. Betton, M. D., M. A. N, S., Fellow of the College of Physicians, of Philadelphia, etc. With the addition of a History of Syphilis, and a complete bibliography and Formulary of Remedies. Collected and arranged, by Paul B. Goddard, M. D. With 50 large quarto plates, comprising 117 beautifully colored illustrations. Philadelphia, 1852. A. Hart, late Carey & Hart; 4to., pp. 359.

This work is eminently French, although not entirely the production of a Frenchman. The introduction is such as we should expect to find in a French book. The history of Syphilis is French; the illustrations are of a character that could be devised and executed in few places other than the French Capital. Dr. Goddard's additions unquestionably enhance the value of the original work, and he is confident that "the finish and coloring"

of the American edition "are fully equal to the original French copy." They are certainly very life-like. We can hardly see how they could be rendered more truthful. As the student looks upon them, he has the disease before him, and not those caricatures which for so long a time passed current in the profession as representatives of Syphilis. Ricord took great pains to secure perfect illustrations, and when he had got them executed he pronounced them "the most graphic and beautiful delineations which have ever appeared in a medical work."

This is a work that to be appreciated must be seen—a notice will give but a poor conception of the materials composing it. It is demonstrative, and the reader must have the illustrations as well as the text under his eye.

It is a costly work, and many practitioners who may be desirous of studying it will find it inconvenient to incur the expense of its purchase; but the plan of forming clubs, which is obtaining more and more in the country, will obviate this difficulty. This great work of Ricord, the highest authority on Syphilitic diseases, will find its appropriate place in all such libraries, and ought to be accessible to all practitioners who are in the way of treating such disorders.

